# Learning curve for bronchial thermoplasty

**DOI:** 10.1111/resp.14248

**Published:** 2022-03-16

**Authors:** Daniel Niewodowski, David Langton

**Affiliations:** ^1^ Department of Thoracic Medicine, Frankston Hospital Peninsula Health Melbourne Victoria Australia; ^2^ Faculty of Medicine, Nursing and Health Sciences Monash University Melbourne Victoria Australia

**Keywords:** bronchial thermoplasty, bronchoscopy, severe asthma, surgical experience

*To the Editors*:

Bronchial thermoplasty (BT) is a novel bronchoscopic treatment which utilizes a radiofrequency catheter, deployed into distal airways, to induce atrophy in the hypertrophied airway smooth muscle layer–a characteristic feature of severe asthma.^1^ Randomized controlled trials and real‐world clinical registries have demonstrated that BT reduces exacerbations, symptoms and medication dependence.^2^ However, BT is unsuccessful in 15%–30% of patients, and the reasons for these failures are unclear.^3^ Many surgical and bronchoscopic procedures (including endobronchial ultrasound) involve a learning curve, where outcomes improve with more procedures and experience.^4^ We assessed if there was an improvement in patient outcomes after BT with increased operator experience.

Data were collected in an Australian tertiary hospital referral centre for asthma. Each patient was under the ongoing care of a specialist respiratory physician, charged with the responsibility of optimizing asthma medication, medication adherence and treatment of comorbidities prior to any evaluation for BT. Patients were not considered for BT unless they met the European Respiratory Society/American Thoracic Society (ERS/ATS) definition of severe asthma, and were poorly controlled despite receiving triple inhaler therapy. All patients underwent HRCT scanning as part of the initial baseline assessment, and patients considered to have radiological evidence of emphysema did not proceed to BT.

The first 50 patients undergoing BT at this centre were included in the analysis—there were no exclusions. Procedures were performed between 2014 and 2020, by a single operator with over 25 years' experience in bronchoscopy, but no previous experience with BT. All bronchoscopies were performed under general anaesthesia, using the Alair Bronchial Thermoplasty System (Boston Scientific, NSW, Australia). Prednisolone was prescribed for 3 days prior, and for 3 days after each BT procedure. Each patient was electively observed overnight in the hospital, immediately following treatment. The Olympus BF‐Q190 bronchoscope (Olympus Medical Systems, Tokyo, Japan) was used for the first 12 cases, after which the operator switched to using the Olympus BF‐P190 bronchoscope (Olympus Medical Systems, Tokyo, Japan) for all subsequent cases, owing to its smaller outer diameter and hence greater ability to reach more distal airways and facilitate greater radiofrequency treatment.^5^, ^6^

Clinical data were prospectively collected on entry into the registry with assessments at baseline, 6 months and 12 months after BT. Data collected included demography, asthma medication usage, exacerbation history, biomarker data, lung function and the Asthma Control Questionnaire, 5‐item version (ACQ). Exacerbations were defined by the need for an increase in oral corticosteroids for 3 days. Spirometry was undertaken in an accredited laboratory by experienced respiratory scientists, to ERS/ATS standards, using the Jaeger Vyntus Body (Carefusion, Germany) calibrated on the day of patient testing. Predicted values were drawn from the Global Lung Initiative. The number of radiofrequency activations administered during BT was also recorded.

The first 50 patients who underwent BT by this operator were included in this analysis. Patients' outcome data were analysed over time by case number as well as by splitting the cohort into half and comparing the first 25 patients with the second 25 patients. The baseline characteristics of these patients are detailed in Table 1.

**TABLE 1:** Baseline characteristics

	First 25 patients	Second 25 patients	*p*‐values^a^
Age (years)	56.7 ± 13.2	54.9 ± 13.1	0.716
Female sex, *n* (%)	12 (48)	15 (60)	0.570^b^
BMI (kg/m^2^)	29.5 ± 6.8	31.7 ± 6.9	0.212
Smoking (pack‐years)	4.7 ± 9.6	7.7 ± 11.7	0.333
Never smokers (%)	16 (64%)	14 (56%)	0.564
ACQ score	3.2 ± 1.1	3.4 ± 0.8	0.473
FEV1 (% predicted)	55.4 ± 14.8	48.7 ± 17.9	0.153
Exacerbations in 6 months	4.1 ± 3.0	2.9 ± 2.6	0.153
ICS (mcg per day)	1848 ± 693	1720 ± 970	0.493
Prednisolone (mg/day)	6.0 ± 8.8	11.6 ± 14.1	0.080
SABA (puffs per day)	7.5 ± 7.2	12.8 ± 9.1	0.014
Monoclonal antibody, *n* (%)	11 (44)	16 (64)	0.156^b^
Blood eosinophils (cells/μl)	396 ± 316	120 ± 140	0.001
IgE (IU/ml)	126 (253)	40 (175)	0.136^c^
Activations	162 ± 30	196 ± 37	0.004

*Note*: Data are presented as mean ± SD or median (interquartile range).

Abbreviations: ACQ, Asthma Control Questionnaire, 5 item version; FEV1%, prebronchodilator forced expiratory volume in 1 s percentage of predicted; ICS, inhaled corticosteroid dose in beclomethasone equivalence in micrograms per day; SABA, short acting beta‐agonist puffs per day.

^a^ *p*‐values are determined using *t*‐tests, unless otherwise specified.

^b^ *p*‐value is determined using chi‐square test.

^c^ *p*‐value is determined using Mann–Whitney *U*‐test.

Analysis of the baseline data revealed this to be a cohort of very severe asthmatics, with markedly obstructed lung function, low cigarette smoking exposure and frequent steroid‐requiring exacerbations despite high‐dose inhaled steroids and dual long‐acting bronchodilator treatment. The symptom burden was high and accompanied by frequent daily reliever medication usage. The second 25 patients were very similar to the first 25 patients but had greater use of short‐acting beta‐agonists (SABA), higher prednisolone requirement and lower forced expiratory volume in 1 s percentage of predicted (FEV1%), likely due to the associated availability of IL‐5‐directed monoclonal antibodies. The total quantum of radiofrequency treatment administered for each patient increased over time, in line with the deliberate change to the procedural technique.

Overall, the entire cohort of 50 patients had significant improvements from baseline to 12 months after BT in ACQ (3.3 ± 1.0 to 1.9 ± 1.3, *p* <0.001), exacerbation frequency (3.5 ± 2.9 to 1.2 ± 1.9, *p* <0.001), SABA daily usage (10.1 ± 8.7 to 4.7 ± 7.0, *p* <0.001), prednisolone dose (8.8 ± 12.2 to 4.9 ± 7.5, *p* = 0.11) and FEV1% (52.0 ± 16.9 to 58.2 ± 21.3, *p* = 0.004). However, when the outcomes of the first 25 patients were compared to the second 25, there were no significant differences between these same variables. When the changes in each outcome for each patient were examined sequentially, there were no significant changes with increasing operator experience, with almost horizontal regression lines, *r*‐values close to zero and insignificant *p*‐values (Figure 1). If non‐response is defined as the absence of improvement in ACQ by the accepted minimal clinically significant difference of 0.5, then eight of 25 patients in the first cohort (32%) and nine of 25 patients in the second cohort (36%) (*χ* ^2^ = 0.765) would be classified as non‐responders at the 12‐month post BT assessment. There were no significant baseline differences between those who responded to BT and those who did not.

**FIGURE 1:**
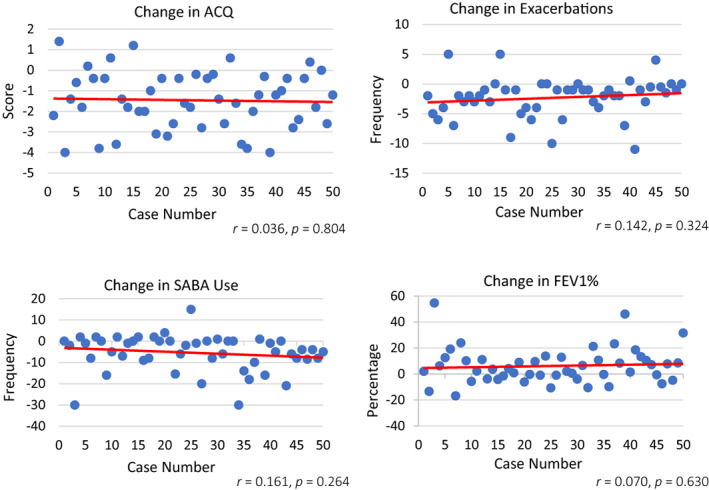
Change in outcome parameters between baseline and 12 months by case number (*n* = 50). ACQ, Asthma Control Questionnaire, 5 item version; exacerbations, oral steroid requiring exacerbations in the previous 6 months; FEV1%, prebronchodilator forced expiratory volume in 1 s percentage of predicted; *r*, Pearson correlation coefficient; SABA, short‐acting beta‐agonist puffs per day

This is the first assessment of the relationship between operator experience and outcomes in BT, and when examined over time there was no evidence of improvement. This observation has important implications, both in terms of learning the procedure and understanding non‐response to BT. The data presented here suggest that proficiency in BT is attained after only a handful of cases, without detectable further improvement in outcomes with increasing experience. This suggests that in the hands of an experienced bronchoscopist, BT is an easy technique to learn. For credentialling purposes, this would mean that only a small number of initial cases might need proctoring before an experienced bronchoscopist could fly solo with BT. The operator was an experienced bronchoscopist, and therefore these conclusions regarding the ease of learning BT could not necessarily be extrapolated to a novice in bronchoscopy. Furthermore, our approach necessitated that the results pertain to a single operator. It would be helpful, and reassuring, to see similar results presented from other centres.

Understanding why some patients do not respond to BT has been an elusive goal. It has not been easy to identify clinical characteristics separating non‐responders from responders. Regression modelling suggests that patients with more severe symptoms, and more frequent exacerbations, are more likely to respond to BT, as noted in the AIR2 trial.^3^, ^7^ Several studies including those by the TASMA group and Ladjemi et al. have suggested that patients with greater eosinophils and IgE as well as an atopic history have better outcomes from BT,^8^, ^9^ but this was not the case in either the North American or Australian BT registries.^3^, ^10^ Overall, response to BT has been described in terms of improvement in asthma symptom questionnaires, such as the ACQ and the Asthma Quality of Life Questionnaire. These are important but subjective measures, and the data presented here show that dichotomizing response based on one parameter alone risks misclassification of some patients.

In conclusion, there was no evidence in our hands that increasing operator experience improved patient outcomes after BT. This implies that BT is an easy technique for the experienced bronchoscopist to learn. Furthermore, it suggests that non‐response to BT corresponds with patient‐related, rather than operator‐related, determinants.

## CONFLICT OF INTEREST

None declared.

## AUTHOR CONTRIBUTION

**Daniel Niewodowski:** Formal analysis (equal); methodology (equal); visualization (equal); writing – original draft (equal); writing – review and editing (equal). **David Langton:** Formal analysis (equal); methodology (equal); visualization (equal); writing – original draft (equal); writing – review and editing (equal).

## HUMAN ETHICS APPROVAL DECLARATION

This study was prospectively approved by the Peninsula Health Human Research and Ethics Committee under the banner of clinical audit. All patients consented for their data to be used.

## Data Availability

The original de‐identified data contained in this paper may be available from the corresponding author upon request and discussion.
